# Glycerine in Veterinary Practice

**Published:** 1890-02

**Authors:** J. Coates


### CASES, SELECTIONS, TRANSLATIONS.

### GLYCERINE IN VETERINARY PRACTICE.

By Dr. J. Coates, M.D., D.V.S.

[From the *American Veterinary Review.*]

I desire to say a few words concerning a class of cases about which a great deal has been written and yet more light is welcome. I refer to flatulent colic. The first months of practice brought many cases of this kind under my observation, and I am free to admit, failure in treatment was more often the rule than the exception. After reading all the literature obtainable on the subject, I decided upon investigating the claims of puncturing the intestines, which was used as a *dernier ressort.*

Having had good success in the first few cases, I adopted it as the only remedy in these cases with a small percentage of deaths, and in the first twelve years operated upon over one thousand cases, with death rate at a minimum.

The one unremitting search of the day is for a key to the true nature of disease, and for remedies which do no violence to natural laws. A large number of popular remedies suffer from the effect of being too generally recommended. According to the claims of their originators, they are useful in the most bewildering variety of ailments, curing every disease. Where too much is claimed, we are apt to grant too little ; and where we have been disappointed by using a drug in cases not suited to it, we are likely to doubt altogether its possession of any therapeutic value.

Within the past few years, the use of glycerine in constipation as a remedial agency has been receiving much attention by practitioners of both human and veterinary medicine. Most of the modern authorities report very enthusiastically in its favor, but none of them have as yet written anything in regard to its use in flatulency, for it is certainly to be regarded as one of the greatest agents.

It is wonderful that in this substance we have, if we use it properly, one of the most potent weapons in the warfare upon flatulent colic.

Having access to a large number of both medical and veterinary journals and pamphlets, my attention was called to an article on the use of rectal injections of pure glycerine in constipation, and I was somewhat dazed that such a prompt action took place. Being always ready to try any new remedy, I have used it on over one hundred dogs for constipation with a prompt action in every case in from one to three minutes ; rarely over two minutes after enema was given. As a firm believer in its use then I tried its effects upon a number of horses, with an action in three or four minutes ; much more readily when the rectum is full of feces. Then I used it on three cases of constipation in horses with poor success, as from my observation the rectum must be partially filled with feces or no action takes place, except the prompt evacuation of the glycerine and a considerable amount of flatus. Acting upon the thought suggested by the fact of the violent expulsion of flatus during its use in these cases, I began using enema of pure glycerine in cases of flatulent colic as an experiment; the results of this method are the best I have ever obtained in the treatment of flatulency, and the most likely to be efficiently carried out.

The only real difficulty in treating cases by puncturing is the objections of the owners, who are afraid some serious result will take place seeing a trocar introduced into the abdominal cavity; but, however admirable this method may be in the treatment of flatulency, if another method can be shown to be as effective, more safe and more simple, the former should give way to the latter. During the past four months I have used it in ten consecutive cases of flatulent colic where the abdominal walls were greatly distended, with the most gratifying results and a complete recovery of each case, and as sufficient time has now elapsed since the treatment of these cases, I report its use for the benefit of the profession, as other members may take it up, for throughout the medical world analysis and research are active and eager for new light. Routine methods and antiquated theories which have only popular prestige and venerable antiquity to sustain them, are no longer competent to satisfy thinking minds.

In the case above mentioned I have found that one ounce of pure glycerine injected into the rectum has caused a prompt and free flow of flatus from the intestinal tract, which is usually kept up for two or three minutes, with a subsidence of the distended abdominal walls. Should there not be a complete collapse of the walls, the same amount of glycerine should be repeated one, two or three times, with intervals of ten or fifteen minutes.

I am not prepared to say how glycerine acts in these cases; probably by reflex action, causing an increased peristaltic action of the large intestines.

Dr. Anacker's experiments on various animals have proven that from half a drachm to half an ounce is sufficient to cause movements of the bowels. Prof. Vogel reports that pure glycerine, or when diluted with one-third of water, when injected into the rectum, causes more or less muscular irritation of the rectum, and produces a remarkably prompt movement. In a few minutes after the injection of glycerine, we can notice, through the action upon the mucous membrane of the rectum, the animal becoming a little uneasy; the anus contracts and dilates spasmodically; the tail raised at times, then arching of the back, followed by defecation. Two or three movements are the average caused, frequently more, and it is claimed, from the condition shown, that they come from the large intestines, and the action is not continued longer than ten minutes. It has been shown that the action of glycerine in constipation is due to its hygroscopic power, drawing the moisture from the tissues of the rectum, slightly irritating the nerves and causing a contraction of the rectum.

The experiments have shown that it does not make much difference about the quantity injected, but the quality. Pure, undiluted, neutral glycerine, specific gravity of 1.225 to 1.235, is the best to use.

Dr. Schindelka has used glycerine on over two hundred and fifty patients (horses, goats, dogs and cats) with positive results in every case. He claims that in young or old, sick or well horses, about eighty drops are sufficient to cause defecation. For cats twenty drops were used and for goats thirty drops.

